# Hunting for dark matter with ultra-stable fibre as frequency delay system

**DOI:** 10.1038/srep11469

**Published:** 2015-07-10

**Authors:** Wanpeng Yang, Dawei Li, Shuangyou Zhang, Jianye Zhao

**Affiliations:** 1 School of Electronics Engineering and Computer Science, Peking University, Beijing, 100871, China

## Abstract

Many cosmological observations point towards the existence of dark-matter(DM) particles and consider them as the main component of the matter content of the universe. The goal of revealing the nature of dark-matter has triggered the development of new, extremely sensitive detectors. It has been demonstrated that the frequencies and phases of optical clock have a transient shift during the DMs’ arrival due to the DM-SM(Standard Model) coupling. A simple, reliable and feasible experimental scheme is firstly proposed in this paper, based on “frequency-delay system” to search dark-matter by “self-frequency comparison” of an optical clock. During the arrival of a dark-matter, frequency discrepancy is expected between two signals with a short time difference(~*ms*) of the same optical clock to exhibit the interaction between atoms and dark-matter. Furthermore, this process can determine the exact position of dark-matter when it is crossing the optical clocks, therefore a network of detecting stations located in different places is recommended to reduce the misjudgment risk to an acceptable level.

One of the most astonishing fundamental observations in cosmology in last decades are the discovery of dark energy and dark matter^1^,^2^,^3^,^4^,^5^,^6^. These two substances of unknown origin comprise about 75% and 20% of the universe’s energy budget^5^. There may be a dark sector, consisting of particles that do not interact with the known forces (strong, weak, or electromagnetic forces), and the existing observations have demonstrated it. Indeed, many dark sectors could exist, with its own intricate structure, distinct particles, and forces. It is often thought that these dark sectors(or “hidden sectors”) may contain new light weakly-coupled particles, particles below the weak-scale that interact with ordinary matter^2^,^7^.

At early cosmological times, very light fields in the initial field configuration could lead to dark-matter via coherent oscillations around the minimum of their potential and/or form non-trivial stable field configurations in physical three-dimensional space if their potential allows such a possibility. These non-trivial stable fields, generally referred as topological defects(TDs), are entwined with spontaneous symmetry breakdown^8^.

As the interaction of TDMs(topological defect dark-matter) with SM can result in some instantaneous changes in some physical properties(eg. mass) of SM elementary particles(e.g. electrons and protons)^9^,^10^, their encounter with the Solar System can lead to observable signatures of dark-matter expressed generically in terms of temporary shifts in the frequencies and phases of optical clocks. Observable effects of TDMs can vary greatly, depending on their mass *m*_*a*_. Some literatures on the subject are supplied^5^, covering a wide range of *m*_*a*_ from 10^−33^ to 10^5^ eV.

In the past two decades, a lot of schemes have been proposed to search DMs, such as the particle physics methods^1^,^3^, the laser and maser interferometry method^4^ and the global network of synchronized atomic magneto-meters method^5^. A novel detecting mechanism based on frequency-delay system is proposed in this letter. The frequency signal which is generated by an optical clock is sent into a frequency-delay system. After a delay time *τ*, the signal is compared with the current generated signal. Optical clocks are the most precise instruments in the world^11^,^12^,^13^, and their frequencies are influenced by the change of fine-structure constant(*α*), they are ideal tools to monitor the change of *α* thereby detecting the arrival of DMs^6^,^11^,^14^,^15^. As to the frequency-delay system, long ultra-stable fibre link can be utilized as it is a highly developed precision frequency-transmitting equipment nowadays^16^. Compared with the reported scheme based on a network of atomic clocks synchronized by high-quality optical fibre links or GPS^6^, our new method possesses two following advantages:

(1) Long-term synchronization over long distance fibre link of different atomic clocks is suffering too many realistic problems. It is complicated to compare the frequency of two independent atomic clocks accurately for a few years. Instead, two frequency signal with short time difference(ms) of the same atomic clock are directly compared in our scheme, which is more feasible and reliable.

(2) Occasional abrupt changes of fibre link and atomic clock systems may result in misjudgment of dark-matter, which can be avoided by the network of detecting stations. However, the synchronization of atomic clocks system could not eliminate such misjudgments because it is unable to determine which clock is effected by TDMs at the exact time.

## Results

**Figure 1:**
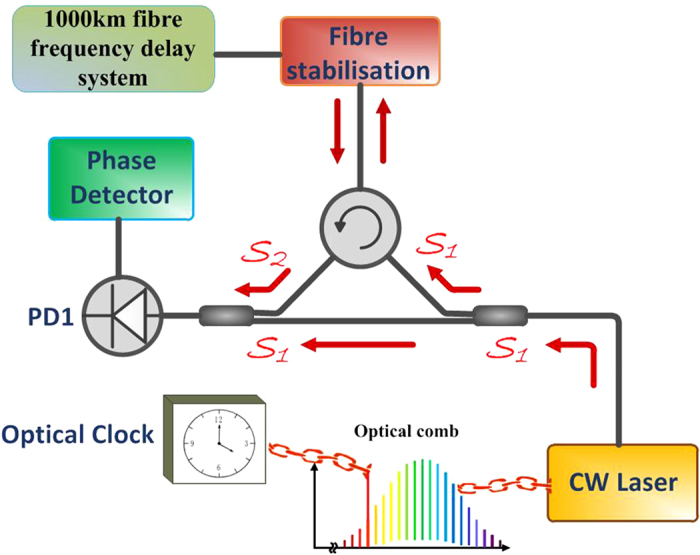
Schematic of the ultra-stable fibre link which is used as a frequency-delay system. Light of a commercial continuous wavelength (CW) fibre laser locked to an optical clock via an optical comb. The CW laser signal is then launched into frequency-delay system. Then frequency-delay system is made up by an ultra stable 1000 km fibre with 10 Er-doped fibre amplifiers to deal with the attenuation.

A schematic of TDs detection is shown in Fig. 1. The continuous wavelength (CW) laser is locked to an optical clock via a optical frequency comb. The light of this laser *S*_1_ = *cos*(*ωt* + *ϕ*) is sent into a frequency-delay system which is made up by a 1000 km ultra-stable fibre. The fibre is stabilized by a fibre stabilization scheme that will be described in detail in **Method Section**. In this method, the slow frequency phase noise below the reverse of the round trip delay time 2 × 10^6^/2 × 10^8^ = 10 *ms* is compensated while the higher frequency phase noise can be controlled by isolating the fibre from the influences such as vibration and so on. The optical power of the transmitted light is boosted by an Er-doped fibre laser (EDFA) every 100 km. After 1000 km the light is retro-reflected with orthogonal polarization by a Faraday mirror. When the retro-reflected light eventually reaches its origin, it has experienced a delay time of *τ* and becomes *S*_2_ = *cos*[*ω*(*t* − *τ*) + *ϕ*]. Then it is mixed with *S*_1_ and the heterodyne signal *S*_*detect*_ = *cos*(*ωτ*) is obtained. When TDs have not come yet, *S*_*detect*_ remains constant. However when TDs come, an extra frequency shift *ω*_*TD*_ is added to the reference signal *S*_1_ due to TDs’ effect on the hyperfine constant *α*. *S*_1_ becomes *cos*[(*ω* + *ω*_*TD*_)*t*]. While the signal *S*_2_ has not been effected by the TDs yet, the detecting signal *S*_*detect*_ is changed into *cos*(*ω*_*TD*_*t* + *ωτ*).The change can be detected and recorded by the phase detector. This change indicates the coming of TDs.

**Figure 2:**
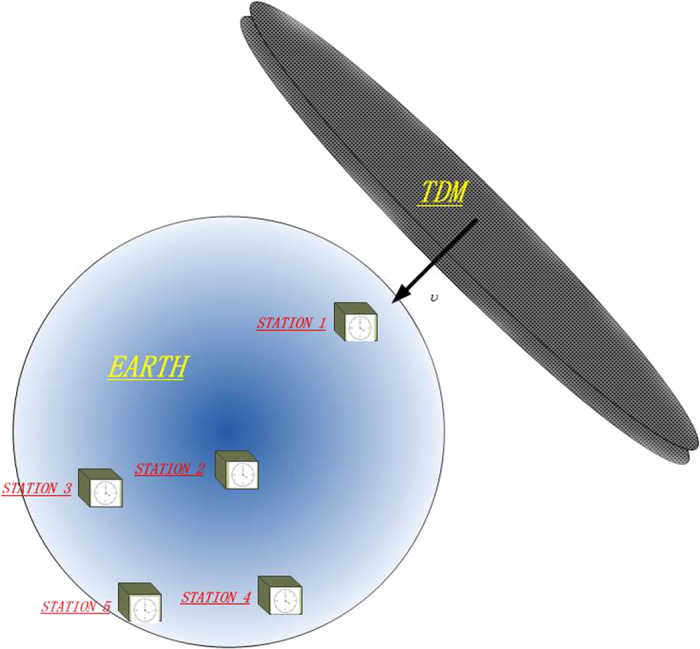
Network of detecting station. The wall-crossing events recorded with the first four detecting station at *t*_*i*_ to determine the normal velocity of the wall, The other station(s) will be used to exclude accidental misjudgments.

Even though the frequency-delay system has been deliberately designed, a particular problem for a search carried out with a single detector is the appearance of brief spikes in the signal caused by the occasional abrupt(e.g, laser-light-mode jumps and sudden fibre vibration). Rejection of these false-positive signals is difficult for a single detector. Therefore, a network composed of more than five detecting stations, illustrated in Fig. 2, is necessary to reduce the probability of misjudgments of TDMs. Four stations will be used for detecting a TDM and determining its geometrical properties. The other stations will serve as cross-check to verify it, based on predicated TDMs’ arrival, a transient frequency shift of the optical clock in a narrow temporal window.

To make our description more specific, an instance of a domain-wall (2D) network^17^ is considered. As shown in Fig. 2, the detecting network is composed of *n* ≥ 5 identical detecting stations (ordinal numbers for 1 to n) separated by distance of *L*_ij_(*i*, *j* ≤ *n*) ≥ 300 km. 3D coordinates of four stations and their encountering moments *t*_1_ ~ *t*_4_ will enable us to unambiguously determine the three-vectors velocity of TDMs. Thus, the narrow window of TDM’s crossing time at the the fifth station, Δ*t* ~ *ms*, can be predicted to exclude accidental misjudgments.

Supposing *τ*_*error*_ and T are corresponding to the average interval time of a single detector misjudgments and the average time between “close encounters” with TDMs. The error decision probability of the detecting network is 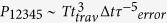, where *t*_*trav*_ ~ *L*_*ij*_/*υ* ~ *s* is the travel time of TDMs from station to station. *P*_12345_ is less than 10^−5^ for *τ*_*error*_ ~ 100 *s* and *T* ≤ 10*yrs*.

Correlation between signals from multiple, geographically separated detectors can be analyzed by the “excess power” statistic method to distinguish them from noise^18^. Moreover, increasing the number of stations in the network and the number of detectors of the stations will decrease the error decision probability and admit shorter interval time of single detector misjudgments^5^. In practice, the network should be composed of the institutions who own optical clocks, such as NIST from U. S., MPI from Germany and SYRTE from France.

## Discussion

In the discussion, a collection of light field beyond SM which can form different types of TDs (monopoles, strings and domain walls) will be introduced. All the light fields are identically considered as *ϕ*, including both scalar field and vector field. The characteristic transverse size of a defect is determined by the field Compton wavelength *d*, *d* ~ *ħ*/(*m*_*a*_*c*). The mass *m*_*a*_ is the typical mass scale of the light field as mentioned above. Only the gross features of TDMs are considered and the amplitude of the field change between inside and outside a TDM is represented as *A*, *A* = *ϕ*_inside_ − *ϕ*_outside_ = *ϕ* (assuming the outside field to be zero).

From the macroscopic view at distance scales much larger than *d*, the TDMs can be characterized by its energy density inside the defect, *ρ*_inside_ ~ *A*^2^/d^2^. The network of TDMs will have an additional distance-scale parameter *L*, the average distance between the defects, which is impossible to calculate without making further assumptions about the mechanisms of TDs formation and evolution.

The energy density of TDM averaged over a large amount of defects is controlled by *ρ*_inside_ and L^6^:


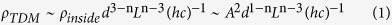


and the average time between “close encounters” with TDs is determined by the galactic velocity of such objects *υ*:


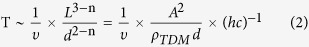


The velocity of galactic objects(*υ*_*g*_) around the Solar System is well known and for the purpose of estimation, it can taken as *υ* ~ *υ*_*g*_ ~ 10^−3^ × c  ≈ 300 *km* ⋅ *s*^−1^.

The maximum energy density of the TDMs in the neighborhood of the Solar System is constrained by the experimental estimated dark-matter energy density^11^, *ρ*_*DM*_  0.4 *G*e*V*/*cm*^3^:


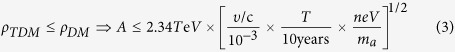


This constraint implies some flexible evolution of the TDMs and the possibility for them to build up their mass inside galaxies. The normalization for L and *m*_*a*_ in equation (3) is suggested by the requirement of having the average time between “close encounters” is within a few years and of having the signal duration in excess of ~*ms*.

The duration of the interaction is related to characteristic transverse size and velocity of the defect:


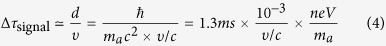


Such a crossing time is comparable to the fibre link delay time and in excess of the optical clock self-frequency comparison response time.

We are also interested in how the fields forming the defect interact with the SM, which can be expressed in the form of the quadratic scalar portal,


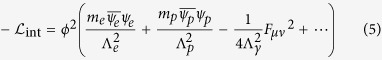


where *m*_*e*,*p*_ and *ψ*_*e*,*p*_ are electron and proton masses and fields, and *F*_*μν*_ are electromagnetic tensor components. The appearance of high-energy scales Λ_*X*_ signifies the effective nature of TDMs, implying that at these scales the scalar portals will be replaced by some unspecified fundamental theory. Equation(5) alter the fundamental constants as follows, respectively,


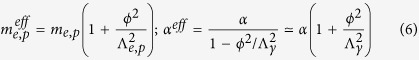


Thus, the instantaneous clock frequency shift may be parameterized as:


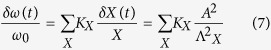


where X runs over the fundamental constants. The dimensionless sensitivity coefficients *K*_*X*_ are known from atomic and nuclear structure calculations^19^.

The time difference between the local frequency signal and returning frequency signal from circular fibre link is to be:


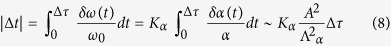


where Δ*τ* is the transmission time of circular fibre link, Δ*τ* ~ *ms* for 200 km fibre link.

The |Δ*t*| and Δ*τ* are combined into a signal factor *S* = |Δ*t*|/Δ*τ* to be directly compared to experimental sensitivity,


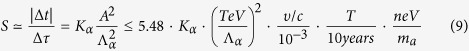


where in the inequality the gravitational constraint from equation (3) is used.

**Figure 3:**
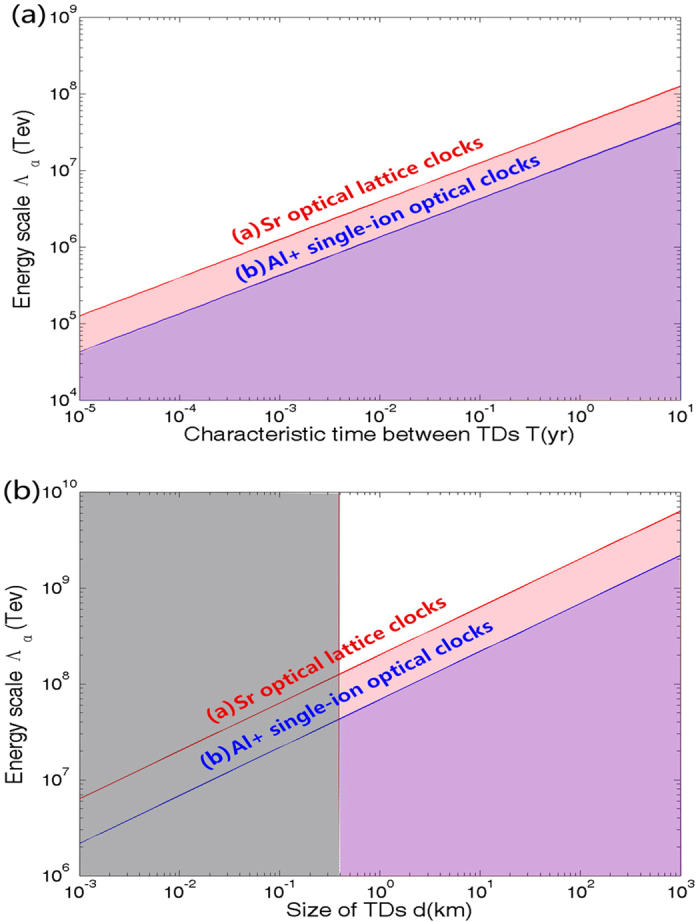
Parameter space open for detection of the TDMs. (**a**) Experimental accessible parameter space in terms of characteristic time T and coupling constraints Λ_*α*_, fixing *m*_*a*_ ~ *neV*, *υ*/*c* ~ 10^−3^. (**b**) Sensitivity curve to the energy scale Λ_*α*_ as a function of the defect size d, fixing *T* ~ 10*years*, *υ*/*c* ~ 10^−3^. The light shared areas under line(a) correspond to the Sr optical lattice clock, and the one under line(b) correspond to the *Al*^+^ single-ion optical clock.

The fractional instability of the clock frequency is characterized by Allan variance *σ*_*y*_(*T*). In order to determine the arrival of TDMs, the signal factor S is required to be satisfied *S* ≥ *σ*_*y*_(Δ*τ*), thus,


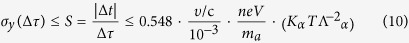


and


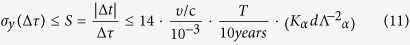


where the unit of *T*, *d*, Λ_*α*_ are taken to be year(yr), km and TeV. In Fig. 3(a), we plot the experimental accessible parameter space in terms of characteristic time between TDMs, *T* = *L*/(10^−3^*c*), and the strengthen of coupling constraints, Λ_*α*_, fixing *m*_*a*_ ~ *neV*, *υ*/*c* ~ 10^−3^. Here we also plot a sensitivity curve to the energy scale Λ_*α*_ as a function of the defect size in Fig. 3(b), fixing the characteristic time between TDMs, *T* ~ 10*yrs*. A Sr optical lattice clock (*K*_*α*_ ~ 0.06,*σ*_(*ms*)_ ~ 2.1 × 10^−17^)^12^ and a Al+ single-ion clock (*K*_*α*_ ~ 0.0078, *σ*_(*ms*)_ ~ 2.3 × 10^−17^)^11^ are considered in Fig. 3(a,b). The light shaded areas under line (a) and line (b) signify the coupling range that can be realistically probed with these two optical clocks and ultra-stabilized fibre link systems. The dark shaded areas in Fig. 3(b) is the region excluded by the requirement of having the signal duration in excess of ~*ms*.

## Methods

**Figure 4:**
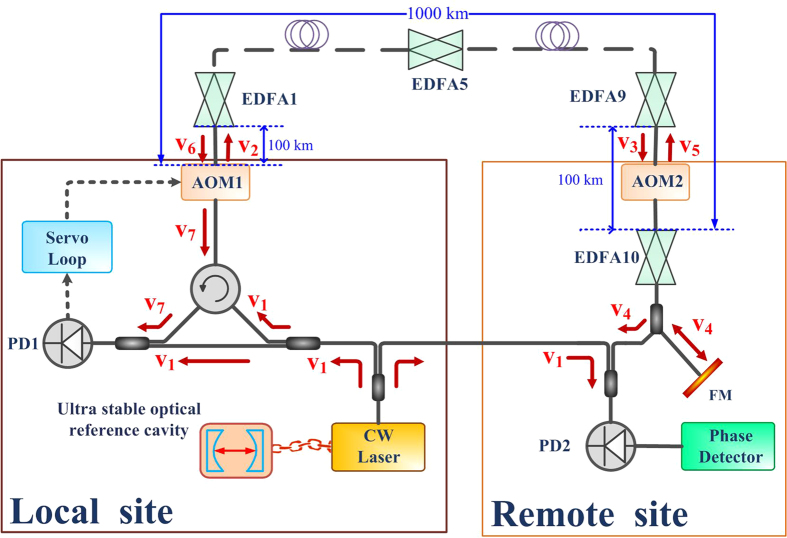
Schematic of the fibre stabilization. Light of a commercial CW fibre laser locked to an ultra stable optical reference cavity. The CW laser signal is then launched into an 1000 km fibre spool with 10 EDFAs to deal with the attenuation. The round-trip light is used to derive an error signal for a servo loop with AOM1 as the actuator. EDFA: erbium-doped fibre amplifier, AOM: acoustic-optic modulator, FM: Faraday mirror, PD: photo diode.

Figure 4 shows a schematic of fibre stabilisation. The scheme shares the same method with that in Ref. [20]. The cw laser is locked to an ultra stable optical reference cavity. The initial signal *V*_1_ = *cos*(*ω*_1_*t* + *ϕ*_0_) is sent into the fibre spool after a phase and frequency shift by an AOM. The signal is changed into *V*_2_ = *cos*(*ω*_1_*t* + *ϕ*_0_ + *ϕ*_*c*_), where *ϕ*_*c*_ is used to actively compensate the fluctuation in the fibre spool which is induced by the environmental impact. An EDFA is placed every 100 km fibre to deal with the attenuation. When the signal arrives the detector end, it has changed into *V*_3_ = *cos*(*ω*_1_*t* + *ϕ*_0_ + *ϕ*_*c*_ − *ϕ*_*p*_), where *ϕ*_*p*_ stands for the phase noise induced by the environmental impact. An AOM (AOM2 in the figure) is induced to distinct the retro-reflected signal with the forward signal. After the AOM2, a constant frequency shift *ω*_*s*_ is induced, and the signal becomes *V*_4_ = *cos*[(*ω*_1_ − *ω*_*s*_)*t* + *ϕ*_0_ + *ϕ*_*c*_ − *ϕ*_*p*_], *V*_4_ is split into two parts. One part is compared with the reference signal *V*_1_, while the rest is reflected back by a faraday mirror. The retro-reflected signal after AOM2 becomes *V*_5_ = *cos*[(*ω*_1_ − 2*ω*_*s*_)*t* + *ϕ*_0_ + *ϕ*_*c*_ − *ϕ*_*p*_] , and after the fibre spool, the signal become *V*_6_ = *cos*[(*ω*_1_ − 2*ω*_*s*_)*t* + *ϕ*_0_ + *ϕ*_*c*_ − 2*ϕ*_*p*_], phase noise *ω*_*p*_*t* + *ϕ*_*p*_ is added. After AOM1, the signal is changed into *V*_7_ = *cos*[(*ω*_1_ − 2*ω*_*s*_)*t* + *ϕ*_0_ + 2*ϕ*_*c*_ − 2*ϕ*_*p*_], *V*_7_ is mixed with *V*_1_ and heterodyne signal *V*_*err*_ = *cos*(2*ω*_*s*_*t* + 2*ϕ*_*p*_ − 2*ϕ*_*c*_) is obtained. The servo loop is used to control *ϕ*_*c*_ − *ϕ*_*p*_ as a constant, hence at the detector end, the heterodyne signal of *V*_1_ and *V*_4_, *V*_*det*_ = *cos*(*ω*_*s*_*t* + *ϕ*_*p*_ − *ϕ*_*c*_) is a constant. Hence, by adjusting the optical length in time, the fibre is stabilised and immune to environment interference.

## Additional Information

**How to cite this article**: Yang, W. *et al.* Hunting for dark matter with ultra-stable fibre as frequency delay system. *Sci. Rep.* **5**, 11469; doi: 10.1038/srep11469 (2015).
